# Senescence and Its Role in Fibrosis

**DOI:** 10.1093/geroni/igaa057.2699

**Published:** 2020-12-16

**Authors:** Claude Le Saux

**Affiliations:** University of California San Francisco, San Francisco, California, United States

## Abstract

The presence of senescent cells (epithelial and mesenchymal) in fibrotic organs has been well established. Removal of senescent cells in animal models of fibrosis indicate an overall beneficial effect. The general consensus is that the senescent cells contribute to the fibrotic phenotype by the secretion of factors, mainly cytokines and chemokines. We recently demonstrated that senescent cells can also secreted eicosanoids. These lipids are implicated in the pathogenesis of fibrosis in multiple organs. Prostaglandins, especially PGE2, are generally regarded as anti-fibrotic, whereas leukotrienes are thought to be pro-fibrotic. Recent studies indicate that the senescence-associated secretory profile is a dynamic process and its composition is cell, tissue, and time-dependent. In this session I will discuss how senescent cells from specific origin have the potential to regulate fibro-genesis and its resolution by switching their eicosanoid profile expression over time. These findings have important implications for emerging senolytic drugs, which have the potential to provide novel therapeutic benefits for the treatment of fibrosis.

